# Curcumin protects against lipopolysaccharide-induced vasoconstriction dysfunction via inhibition of thrombospondin-1 and transforming growth factor-β1

**DOI:** 10.3892/etm.2014.2105

**Published:** 2014-12-03

**Authors:** WEI LU, JIAN-PING JIANG, JUE HU, JUE WANG, MING-ZHI ZHENG

**Affiliations:** 1 Department of Vascular Surgery, Quzhou People’s Hospital, Quzhou, Zhejiang 324000, P.R. China; 2 Department of Clinical Medicine, Zhejiang Medical College, Hangzhou, Zhejiang 310053, P.R. China; 3 Department of Basic Medical Sciences, Zhejiang Medical College, Hangzhou, Zhejiang 310053, P.R. China

**Keywords:** lipopolysaccharide, curcumin, vasoconstriction, thrombospondin-1, transforming growth factor-β1

## Abstract

Sepsis is a complex syndrome characterized by the development of progressive dysfunction in multiple organs. The aim of the present study was to investigate the protective effect of curcumin against lipopolysaccharide (LPS)-induced vasoconstrictive dysfunction, and to investigate the possible underlying mechanism. Male Sprague-Dawley rats were randomly divided into the following groups: Control, sepsis and curcumin. A sepsis model was established by an intraperitoneal (i.p.) injection of 5 mg/kg LPS. Thoracic aortic rings obtained from the rats were mounted in an organ bath and the vasoconstriction of the rings was recorded. In addition, the serum E-selectin levels were determined by an enzyme-linked immunosorbent assay. The expression levels of thrombospondin (TSP)-1 and transforming growth factor (TGF)-β1 in the aortic tissue were detected by immunohistochemistry. Vasoconstriction of the aortic rings was found to significantly decrease in the sepsis rats when compared with the control group. However, curcumin (10 or 20 mg/kg, i.p.) prevented the vasoconstrictive dysfunction induced by LPS. The serum level of E-selectin and the expression levels of TSP-1 and TGF-β1 significantly increased in the sepsis rats when compared with the control group rats; however, the levels decreased significantly following treatment with curcumin (10 or 20 mg/kg). Furthermore, hematoxylin and eosin staining revealed that curcumin alleviated the LPS-induced damage in the aortic tunica intima and tunica media. Therefore, the results indicated that curcumin alleviates LPS-induced vasoconstrictive dysfunction in the thoracic aorta of rats. In addition, the inhibition of TSP-1 and TGF-β1 expression may be involved in the mechanism underlying this protective effect.

## Introduction

Sepsis is a serious health risk, which may ultimately lead to the failure of multiple organs, including the liver, lungs, cardiovascular system, kidneys and gastrointestinal tract (1–3). In animal models, vascular hyperactivity to vasoconstrictor agents, induced by bacterial lipopolysaccharide (LPS), is an important factor contributing to the eventual circulatory failure (4).

Curcumin, which is derived from the rhizome of the *Curcuma longa* (turmeric) plant, has been widely used in South East Asia as a food component, but also in the treatment of numerous diseases (5,6). Curcumin has been shown to possesses anti-inflammatory and antioxidative properties (7). In addition, curcumin has been reported to inhibit LPS-induced endothelium barrier disruption by increasing endothelium barrier integrity and inhibiting LPS-mediated nuclear factor (NF)-κB activation and tumor necrosis factor (TNF)-α production (8). However, whether curcumin is able to protect against LPS-induced vascular hypoactivity has not been fully investigated.

Thrombospondin (TSP)-1 is found in platelet β-granules and is produced by a number of cell types, including endothelial cells and vascular smooth muscle cells (9). It has been reported that the expression of TSP-1 is increased on the platelet surface in patients suffering from sepsis, and that polymorphisms of the TSP-1 gene are associated with the progression of sepsis-related organ failure (10). Transforming growth factor (TGF)-β1 is a cytokine that plays a pivotal role in the regulation of the immune/inflammatory response and subsequent tissue repair processes. TGF-β1 has been associated with a number of human diseases; for example, functioning as a promoter of fibrosis (11). Numerous studies have shown that TSP-1 is responsible for a significant proportion of TGF-β1 activation in a variety of organs, including the lungs, pancreas, kidneys, liver and heart (12,13). However, the exact roles of TSP-1 and TGF-β1 in sepsis-associated vasoconstrictive dysfunction remain unknown. In addition, whether TSP-1 and TGF-β1 are involved in the protective effect of curcumin on LPS-induced vasoconstrictive dysfunction is not clear. In the present study, a LPS-induced sepsis rat model was used to investigate the association between the protective effects of curcumin and the alterations in TSP-1 and TGF-β1 expression.

## Materials and methods

### Reagents

LPS (from *Escherichia coli* 055:B5), curcumin, phenylephrine (PE) and acetylcholine (ACh) were obtained from Sigma-Aldrich (St. Louis, MO, USA). DMSO was purchased from Jingchun Biological Technology Co., Ltd. (Shanghai, China). TSP-1 and TGF-β1 antibodies were purchased from Santa Cruz Biotechnology, Inc. (Dallas, TX, USA). A rat E-selectin enzyme-linked immunosorbent assay (ELISA) kit was purchased from Shanghai Yuanye Biological Technology Co., Ltd. (Shanghai, China). Krebs-Henseleit (KH) solution compositions were as follows (in mmol/l): NaCl, 120; NaHCO_3_, 25; MgSO_4_, 1.2; CaCl_2_, 1.25; KCl, 4.5; KH_2_PO_4_, 1.2; and glucose, 11.1 (pH 7.4).

### Animal preparation

A total of 60 male Sprague-Dawley rats (weight, 220–250 g; age, 40–50 days) were obtained from the Experimental Animal Center of Zhejiang Academy of Medical Sciences (Zhejiang, China), and cared for in compliance with the Guide for the Care and Use of Laboratory Animals (National Institutes of Health, Bethesda, MA, USA; 14). All experimental protocols were approved by the Ethics Committee on Animal Experimentation of Zhejiang Academy of Medical Sciences. The rats were randomly divided into six groups, including the control, LPS [intraperitoneal (i.p.) injection of 5 mg/kg LPS in sterile saline], LPS + curcumin [i.p. injection of 5 mg/kg LPS and 5, 10 or 20 mg/kg curcumin in 10% dimethyl sulfoxide (DMSO)] and curcumin groups (i.p. injection of 20 mg/kg curcumin in 10% DMSO). The control and LPS groups also received an equal amount of 10% DMSO.

### Preparation of aortic rings and measurement of vasoconstriction

Aortic rings were prepared using the method described by Fang *et al* (7). Briefly, 24 h after the i.p. injection of the respective reagents, all the rats were anesthetized with 3% pentobarbital sodium (60 mg/kg). Then thoracic cavity was opened and the thoracic aorta was surgically removed. The thoracic aorta was cut into 2–3-mm rings, and mounted in an organ bath system (Nanjing MedEase Technology Co., Ltd., Nanjing, China) containing KH solution (95% O_2_ and 5% CO_2_, 37°C). Isometric tension was recorded using a data-acquisition system (Nanjing MedEase Technology Co., Ltd.). After 60 min of equilibration at a passive tension of 2 g, the rings were treated three times with KCl (60 mmol/l) to evoke maximal excitability. The integrity of the vascular endothelium was examined by adding PE (10^−6^ mol/l) to the organ bath, followed by the addition of ACh (10^−5^ mol/l). Only tissues that responded to ACh with a >70% reduction of PE-induced vasoconstriction were considered to have an intact endothelium. After washing, PE (10^−8^-3×10^−6^ mol/l)-induced vasoconstriction was recorded. Contractile responses were expressed as the percentage of the maximum response induced by KCl initially (15). The maximum effect (E_max_) and the concentration eliciting 50% of E_max_ (EC_50_) were obtained from the cumulative concentration-response curve of PE. Subsequently, pD_2_ was calculated as −logEC_50_.

### Measurement of serum E-selectin levels

Peripheral rat blood was collected in a tube with coagulant, and centrifuged at 2,200 × g for 10 min at 4°C. Serum supernatant was stored at −80°C until required for analysis. The levels of E-selectin in the serum samples were quantitated using an ELISA, according to the manufacturer’s instructions. E-selectin was determined spectrophotometrically by Tecan Infinite M200 (Tecan Group Ltd., Mannedorf, Switzerland) at an absorbance wavelength of 450 nm.

### Immunohistochemistry and histology

Experiments were performed on slices from 10% formalin-fixed tissue embedded in paraffin. Slices were incubated with commercial primary antibodies against TSP-1 (mouse IgG; 1:200; cat. no. sc-59887; Santa Cruz Biotechnology, Inc., Dallas, TX, USA) or TGF-β1 (rabbit IgG; 1:200; cat. no. sc-146; Santa Cruz Biotechnology, Inc.) overnight at 4°C, followed by a horseradish peroxidase-conjugated secondary antibody (1:500; goat anti-mouse IgG, cat. no. sc-2005 or; goat anti-rabbit IgG, cat. no. sc-2004; Santa Cruz Biotechnology, Inc.) at room temperature. Peroxidase activity was visualized with 3,3′-diaminobenzidine and the slices were subsequently counterstained with hematoxylin. Each slice was observed in ten randomly selected fields, and the percentage of TSP-1-positive or TGF-β1-positive cells in the total cells was calculated. Hematoxylin and eosin (HE) staining was also performed for morphometric analysis.

### Statistical analysis

Data are expressed as the mean ± standard error of the mean, and were analyzed by two-way analysis of variance (ANOVA) with a Bonferroni post hoc test or one-way ANOVA with a Newman-Keuls post hoc test, using Prism 5.0 (GraphPad Software, Inc., La Jolla, CA, USA). P<0.05 was considered to indicate a statistically significant difference.

## Results

### Effects of LPS on PE-induced vasoconstriction in aortic rings

**Figure 1:**
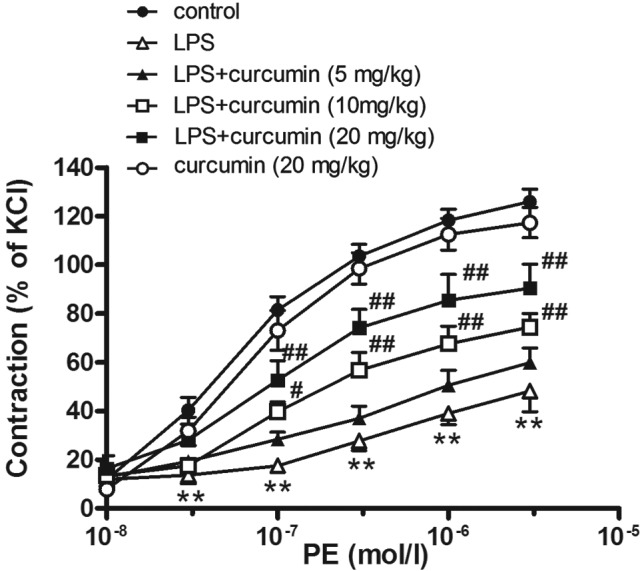
Effect of curcumin on the cumulative dose-response curves to PE in aortic rings of sepsis rats. Data are expressed as the mean ± standard error of the mean (n=8). ^**^P<0.01, vs. control; ^#^P<0.05 and ^##^P<0.01, vs. LPS. PE, phenylephrine; LPS, lipopolysaccharide.

Compared with the control group, the injection of LPS decreased the PE-induced vasoconstriction in the thoracic aortic rings (LPS group: E_max_, 54.3±0.8% and pD_2_, 6.29±0.29; vs. control group: E_max_, 126.0±0.5% and pD_2_ 7.29±0.10; P<0.01; Fig. 1).

### Effects of curcumin on LPS-induced contractile dysfunction in aortic rings

Curcumin (10 or 20 mg/kg) was shown to partly reverse LPS-induced contractile dysfunction (E_max_, 76.4±4.8 and 93.0±6.9%, respectively; pD_2_, 6.92±0.18 and 7.05±0.23%, respectively; P<0.05). However, a low concentration of curcumin (5 mg/kg) had no effect on PE-induced vasoconstriction in sepsis rats. Furthermore, curcumin (20 mg/kg) alone had no effect on PE-induced vasoconstriction in the aortic rings (E_max_, 119.8±4.7%; pD_2_, 7.25±0.12; P>0.05, vs. control group; Fig. 1).

### Effects of curcumin on the LPS-induced increase in E-selectin levels

**Figure 2:**
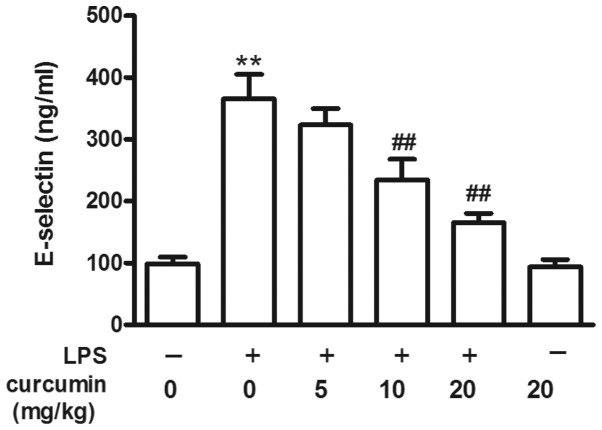
Changes in the levels of serum E-selectin in sepsis rats. Data are expressed as the mean ± standard error of the mean (n=8). ^**^P<0.01, vs. control; ^##^P<0.01, vs. LPS. LPS, lipopolysaccharide.

Compared with the control group, LPS increased the serum E-selectin levels (P<0.01; Fig. 2). However, curcumin (10 or 20 mg/kg) prevented the LPS-induced increase in serum E-selectin levels, although low concentrations of curcumin (5 mg/kg) had no protective effect. Curcumin (20 mg/kg) alone had no effect on serum E-selectin levels when compared with the control group (P>0.05; Fig. 2).

### Effects of curcumin on LPS-induced TSP-1 and TGF-β1 overexpression

**Figure 3:**
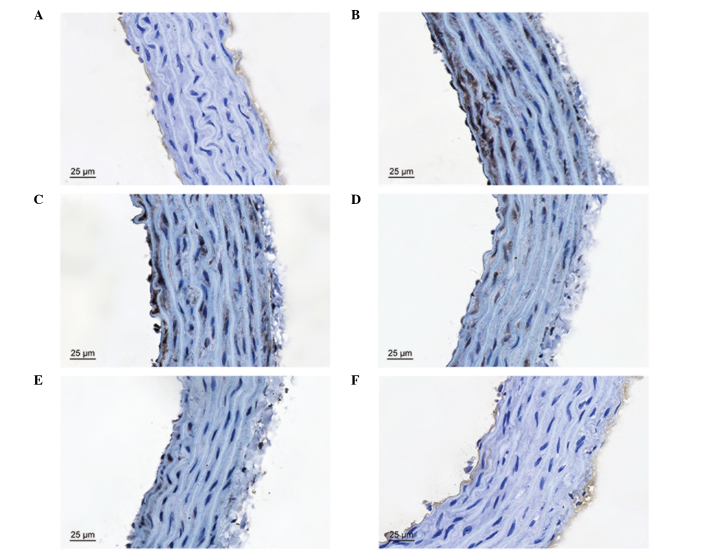
Immunohistochemical results of TSP-1 protein expression in the thoracic aortic tissues of rats in the (A) control, (B) LPS, (C) LPS + curcumin (5 mg/kg), (D) LPS + curcumin (10 mg/kg), (E) LPS + curcumin (20 mg/kg) and (F) curcumin (20 mg/kg) groups. TPS, thrombospondin; LPS, lipopolysaccharide. The comparison of TSP-1 expression in the thoracic tissue (claybank spots) in the cytoplasm represents positive staining.

**Figure 4:**
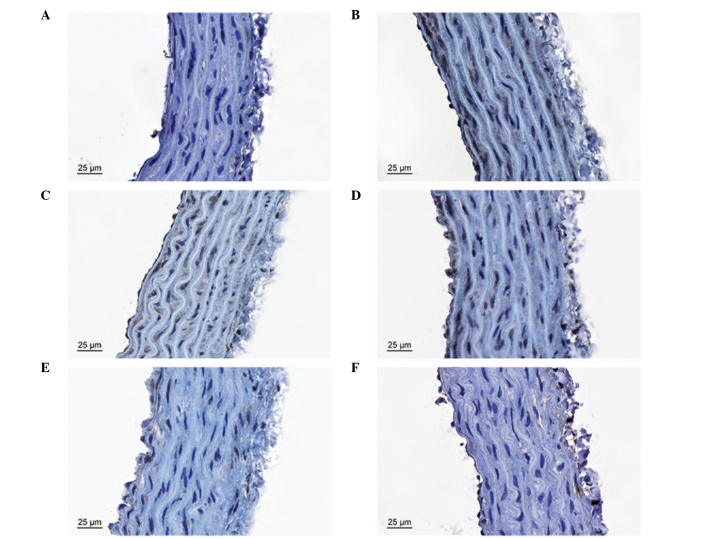
Immunohistochemical results of TGF-β1 protein expression in the thoracic aortic tissues of rats in the (A) control, (B) LPS, (C) LPS + curcumin (5 mg/kg), (D) LPS + curcumin (10 mg/kg), (E) LPS + curcumin (20 mg/kg) and (F) curcumin (20 mg/kg) groups. TGF, transforming growth factor; LPS, lipopolysaccharide. The comparison of TSP-1 expression in the thoracic tissue (claybank spots) in the cytoplasm represents positive staining.

**Figure 5:**
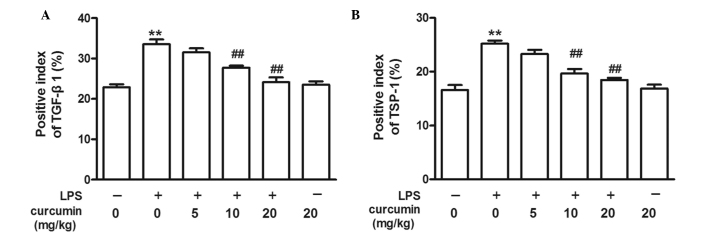
Analysis of (A) TGF-β1-positive and (B) TSP-1-positive cells in sepsis rats. Data are expressed as the mean ± standard error of the mean (n=8). ^**^P<0.01, vs. control; ^##^P<0.01, vs. LPS. TGF, transforming growth factor; TSP, thrombospondin; LPS, lipopolysaccharide.

TSP-1 and TGF-β1 immunoreactivity were observed as a granular immunostaining pattern in the vascular tissues. In the control group, TSP-1- and TGF-β1-positive cells were observed in the vascular endothelium and subendothelial layer, but not in the smooth muscle cells. LPS increased the percentage of TSP-1- and TGF-β1-positive cells in the vascular endothelium and subendothelial layer, and positive cells were also observed in the smooth muscle cells. Thus, treatment with curcumin (10 or 20 mg/kg) inhibited LPS-induced TSP-1 and TGF-β1 overexpression (Figs. 3–5).

### Effects of curcumin on LPS-induced vascular injury

**Figure 6:**
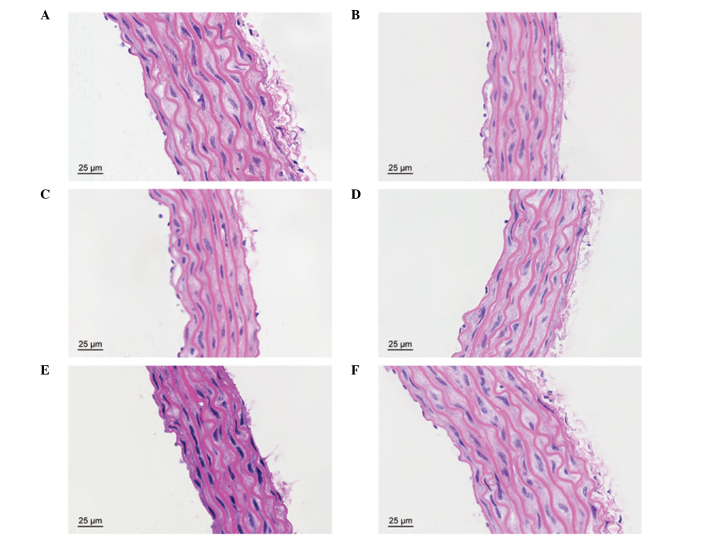
Hematoxylin and eosin staining of the thoracic aortic tissues of rats in the (A) control, (B) LPS, (C) LPS + curcumin (5 mg/kg), (D) LPS + curcumin (10 mg/kg), (E) LPS + curcumin (20 mg/kg) and (F) curcumin (20 mg/kg) groups. LPS, lipopolysaccharide.

In the control group, the vascular structure of each layer was clear and complete. However, the thoracic aortic structure was evidently damaged by LPS, particularly in the endothelium, subendothelial layer and elastic membrane. Treatment with curcumin was shown to protect against LPS-induced vascular injury (Fig. 6).

## Discussion

Previous studies have shown that patients who survive severe sepsis have an increased risk of cardiovascular events (16), and sepsis-induced cardiovascular dysfunction is one of the major causes of mortality in sepsis patients (17,18). However, whether aortic contractile function is injured during sepsis remains unclear. The present study demonstrated that PE-induced vasoconstriction of the aortic rings exhibited a significant decline following treatment with LPS for 24 h, suggesting that sepsis may cause aortic vasoconstrictive dysfunction. Previous studies have indicated that a K^+^ channel abnormality may be an underlying mechanism (4,18). In the present study, LPS was also demonstrated to increase the level of serum E-selectin, which is consistent with results reported by Kim *et al* (8). E-selectin, which can be synthesized by vascular endothelial cells, has the role of promoting leukocyte rolling along the vascular endothelium. Under normal physiological conditions, there is very little or almost no expression of E-selectin in vascular endothelial cells. LPS is known to activate innate immune responses, resulting in the production of a great variety of inflammatory cytokines (19). Particular *in vitro* and *in vivo* stimuli, including inflammatory cytokines (TNF-α) or activated transcription factors (NF-κB), can induce vascular endothelial cells to overexpress E-selectin (20,21). E-selectin molecules on the cell membrane surface may detach or dissolve, becoming soluble (22). Serum soluble E-selectin is an important index of vascular endothelial cell injury (23). Thus, the results of the present study indicate that LPS may lead to aortic endothelial injury.

Curcumin, a natural yellow pigment, is derived from the rhizome of *Curcuma longa* (24). The compound has been used in traditional Chinese medicine for centuries to treat a variety of diseases, including pulmonary fibrosis, diabetic nephropathy and cervical cancer (25–27). The results of the present study demonstrated that curcumin may have a preventative effect against sepsis-induced vasoconstrictive dysfunction and the increase in serum E-selectin levels. Furthermore, the results of HE staining indicated that curcumin decreased the pathological changes of the aortic vascular tissues induced by sepsis; however, the specific mechanism underlying these effects remains unknown.

TSP-1 is a matrix glycoprotein with diverse roles in various cellular and physiological processes (28). Sources of TSP-1 include activated platelets, leukocytes, endothelial cells, vascular smooth muscle cells and fibroblasts (29–31). The expression of TSP-1 is usually increased at sites of inflammation and is hypothesized to play an important role in multiple biological processes, including thrombosis, wound healing and the immune response (32–36). In animal models of diabetes, TSP-1 expression was found to increase significantly in the cavernous tissue (37). TSP-1 expression may impact the prognosis of patients with severe sepsis by inhibiting innate immune function. Furthermore, TSP-1 deficiency has been reported to have a protective effect in cecal ligation puncture and *Escherichia coli* injection (i.p.) models of peritoneal sepsis (38). The present study showed that the protein expression of TSP-1 increased in the aortic tissues of LPS-treated rats, while curcumin was able to inhibit this overexpression.

TGF-β1 is generally considered to be involved in the regulation of inflammation and tissue remodeling following injury (39–41). In normal blood vessels, TGF-β1 inhibits endothelial and vascular smooth muscle cell proliferation (42,43). TSP-1 has been reported as an important activator of TGF-β1 in diabetic nephropathy. KRFK sequences in TSP-1 have been shown to combine with the latency-associated peptide (LAP) region of latent TGF-β1, inducing changes in the spatial configuration of the LAP. Subsequently, the receptor binding site on TGF-β1 is exposed and the TSP-1/TGF-β1 complex is activated (13). In addition, TSP-1 has been shown to upregulate latent TGF-β1 activation, and in turn, TGF-β1 may facilitate TSP-1 synthesis (44). LPS can strongly induce an increase in the expression of TGF-β1 and acute phase proteins in TGF-β1-transgenic mice (45). Sepsis promotes the release and activity of TGF-β1, thereby causing alveolar epithelial cell dysfunction (46). Furthermore, previous studies have reported that curcumin protects against sepsis-induced acute lung injury by inhibiting the expression of molecules involved in the TGF-β1/SMAD3 pathway (47,48). In keloid fibroblast cells, the excessive production of extracellular matrix may be blocked and/or rapidly decreased by curcumin through depression of the TGF-β1/SMAD pathway (49). In the present study, the protein expression of TGF-β1 increased in the aortic tissues during sepsis and curcumin was shown to inhibit this sepsis-induced overexpression, indicating that TGF-β1 may be involved in the protective effect of curcumin against sepsis-induced vasoconstriction injury.

In summary, the present study has provided experimental evidence demonstrating that curcumin alleviates LPS-induced vasoconstrictive dysfunction in rat thoracic aortas. In addition, inhibition of TSP-1 and TGF-β1 expression may be involved in this protective effect.
